# Update on the Treatment of Metastatic Squamous Non-Small Cell Lung Cancer in New Era of Personalized Medicine

**DOI:** 10.3389/fonc.2017.00050

**Published:** 2017-03-27

**Authors:** Sara Victoria Soldera, Natasha B. Leighl

**Affiliations:** ^1^Division of Medical Oncology, Princess Margaret Cancer Centre, Toronto, ON, Canada

**Keywords:** targeted therapy, personalized medicine, lung cancer, squamous cell carcinoma, molecular sequence data

## Abstract

Despite advances in molecular characterization and lung cancer treatment in recent years, treatment options for patients diagnosed with squamous cell carcinoma of the lung (SCC) remain limited as actionable mutations are rarely detected in this subtype. This article reviews potential molecular targets and associated novel agents for the treatment of advanced SCC in the era of personalized medicine. Elements of various pathways including *epidermal growth factor receptor, PI3KCA, fibroblast growth factor receptor, retinoblastoma, cyclin-dependent kinases, discoidin domain receptor tyrosine kinase 2*, and *mesenchymal-to-epithelial transition* may play pivotal roles in the development of SCC and are under investigation for drug development.

## Introduction

**Figure 1:**
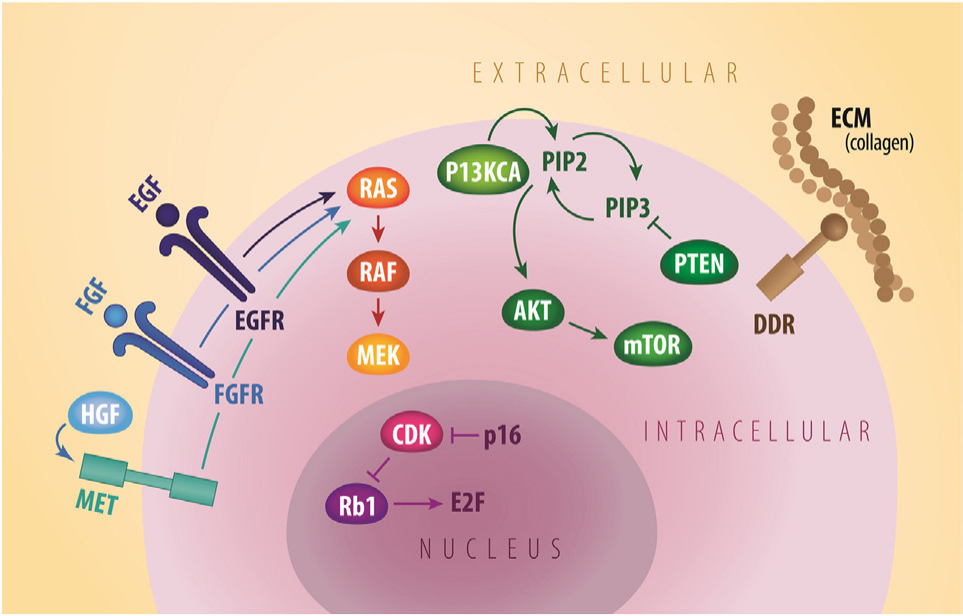
**General signaling schema of cell membrane (EGFR, FGFR, MET, and DDR2), cytoplastic (PI3KCA, AKT, mTOR, and PTEN), and nuclear (Rb1 and CDK) molecular targets in squamous NSCLC**. CDK, cyclin dependent kinases; DDR2, discoidin domain receptor tyrosine kinase 2; ECM, extracellular matrix; EGF, epidermal growth factor; EGFR, epidermal growth factor receptor; FGF, fibroblast growth factor; FGFR, fibroblast growth factor receptor; HGF, hepatocyte growth factor; mTOR, mammalian target of rapamycin; MET, mesenchymal-to-epithelial transition; PTEN, phosphatase and tensin homolog. Credit to Matthew Villagonzalo, graphic artist, University Health Network.

In 2016, lung cancer remains the most commonly diagnosed malignancy and accounts for the most cancer-related deaths worldwide, representing a significant global health burden (1). The majority of these neoplasms are pathologically categorized as non-small cell lung cancer (NSCLC), which is further divided into three main pathological subtypes: adenocarcinoma, squamous cell carcinoma (SCC), and large cell carcinoma. SCC represents an estimated 20% of NSCLC in developed countries and is mainly attributed to tobacco consumption (2). In the past decade, breakthroughs in molecular characterization of cancers have revolutionized the classification and therapeutic arsenal for lung malignancies. With the discovery of oncogenic driver mutations in epidermal growth factor receptor (EGFR) and rearrangements in *anaplastic lymphoma kinase* (*ALK*) and *ROS1*, there has been a paradigm shift from a “one size fits all” approach to lung cancer treatment to more precise and rational targeted therapy (3, 4). Targeted agents such as EGFR and ALK tyrosine kinase inhibitors (TKI) are now routinely used in clinical practice and have contributed to improving the previously dismal prognosis of this malignancy (5–12). Unfortunately, the impact of these developments to date is largely limited to lung adenocarcinoma as these actionable mutations are rarely detected in other subtypes such as pure SCC (13). This article reviews potential molecular targets and associated novel treatments for advanced lung SCC in the new era of personalized medicine (Figure 1; Table 1).

**Table 1:** **Estimated incidence of targetable molecular aberrations in squamous non-small cell lung cancer (NSCLC)**.

Gene and aberration	Incidence (%)	Reference
**EGFR**		
Mutation	0–4.9	Lindeman et al. (13)
	1.1	TCGA (14)
	4	Spoerke et al. (15)
Amplification	7	TCGA (14)
**ALK**		
Rearrangement	0	Lindeman et al. (13)
**FGFR**		
Mutation	0.8^b^	CLCGP/NGM (16)
	8^c^	TCGA (14)
Amplification	9.7–22	Weiss et al. (17)
	16^e^	Heist et al. (18)
**PI3KCA**		
Amplification	37	Spoerke et al. (15)
	33	Yamamoto et al. (19)
Mutation	9	Spoerke et al. (15)
	16	TCGA (14)
	3.6	Yamamoto et al. (19)
	6.5	Kawano et al. (20)
**PTEN**		
Loss	21	Spoerke et al. (15)
Mutation	8	TCGA (14)
	10.2	Jin et al. (21)
**Rb1**		
Mutation	7	TCGA (14)
**CDK**		
Amplification^d^	Significantly amplified	TCGA (14)
**CDKN2A**		
Mutation	15	TCGA (14)
Loss^a^	72	TCGA (14)
**DDR2**		
Mutation	1.1	CLCGP/NGM (16)
	3.8	Hammerman et al. (22)
**MET**		
amplification	6.2–10.3	Go et al. (23)

*^a^Via epigenetic silencing by methylation, inactivating mutation, exon 1β skipping and homozygous deletion*.

*^b^All FGFR3 mutations*.

*^c^FGFR1, 2, 3, and 4 mutations*.

*^d^Significant amplification of CDK6 and CCND1*.

*^e^FGFR1 amplification*.

In recent years, comprehensive molecular profiling of SCC has revealed that these cancers harbor numerous genomic and epigenomic alterations with a reported mean of 360 exonic mutations, 165 rearrangements, and 323 segments of copy-number alteration per tumor (14). Relative to other tumor types, only malignant melanomas contain a higher burden of genetic abnormalities (24). This is not surprising since both of these cancers are associated with significant exposure to carcinogens. In fact, SCC is known to be strongly associated with chronic tobacco exposure (25). With such a complex genetic landscape and associated high immunogenicity, this tumor type has been an interesting target for immunotherapy and chemotherapy, but the development of targeted agents has thus far represented a significant challenge (26). To address this lack of targeted therapies, the Cancer Genome Atlas Project compared SCC samples to normal pulmonary tissue in order to identify potential actionable mutations (14). Eleven recurrent genomic abnormalities were reported, including *tumor protein 53, cyclin-dependent kinase inhibitor 2A* (*CDKN2A*), *phosphatase and tensin homolog* (*PTEN*), *PIK3CA, Kelch-like ECH-associated protein 1, mixed-lineage leukemia protein 2, human leukocyte antigens A, nuclear factor erythroid-derived 2-like 2, NOTCH1*, and *retinoblastoma (Rb1)* (Figure 1; Table 1). Aberrations in these genes are thought to promote oncologic transformation and progression through their effect on cell survival and proliferation, cell cycle progression, metastatic spread, genetic instability, and response to oxidative stress. Other series have demonstrated similar recurring mutations, while also demonstrating significant abnormalities in *Kirsten rat sarcoma viral oncogene homolog* (*KRAS*), *PI3KCA, mesenchymal-to-epithelial transition (MET), human epidermal growth factor receptor 2, fibroblast growth factor receptor (FGFR), platelet-derived growth factor receptors* (*PDGFR*), *BRAF*, and *discoidin domain receptor tyrosine kinase 2 (DDR2)* (15–23, 27) (Figure 1; Table 1). These findings have fueled the development of multiple targeted agents directed against these pathways (Table 2).

**Table 2:** **Clinical trials of targeted therapies in squamous NSCLC**.

Agents	Trial	Phase	Outcome	Reference
			(95% CI)	
**EGFR**				
Erlotinib versus placebo	BR21	III	OS HR 0.70 (0.58–0.85)	Shepherd et al. (28)
Gefitinib versus D	INTEREST	III	OS HR 1.020 (0.905–1.150)	Kim et al. (29)
Afatinib versus erlotinib	LUX-Lung 8	III	PFS HR 0.81 (0.69–0.96)	Soria et al. (30)
			OS HR 0.81 (0.69–0.95)	
C + T ± cetuximab	BMS 099	III	PFS HR 0.902 (0.761–1.069)	Lynch et al. (31)
			OS HR 0.890 (0.754–1.051)	
Cis + V ± cetuximab	FLEX	III	OS HR 0.871 (0.762–0.996)	Pirker et al. (32)
Chemo ± cetuximab	Pujol et al.	Individual patient data meta-analysis	PFS HR 0.90 (0.82–1.00)	Pujol et al. (33)
			OS HR 0.88 (0.79–0.97)	
Cis + G ± necitumumab	SQUIRE	III	OS HR 0.84 (0.74–0.96)	Thatcher et al. (34)
P ± matuzumab (1 versus 3 week)	Schiller et al.	Randomized II	ORR 5 versus 11% (*p* = 0.332)^a^	Schiller et al. (35)
			OS 1 week HR 0.67 (0.3–0.21)	
			OS 3 week HR 1.66 (0.9–0.86)	
C + T ± panitumumab	Crawford et al.	Randomized II	TTP HR 0.9 (0.66–1.21)	Crawford et al. (36)
**FGFR**				
D ± nintedanib	LUME-lung 1	III	PFS HR 0.79 (0.68–0.92)	Reck et al. (37)
			OS HR 0.94 (0.83–1.05)	
Dovitinib	Lim et al.	Single arm II	ORR 11.5% (0.8–23.8)	Lim et al. (38)
AZD4547	Paik et al.	Ib	0 CR, 1 PR, 4 SD, 9 PD^b^	Paik et al. (39)
BGJ398	Nogova et al.	I	15.4% PR, 34.6% SD	Nogova et al. (40)
			23.1% PR, 26.9% unknown	
**PI3KCA**				
Everolimus	Soria et al.	Single arm II	ORR 4.7%	Soria et al. (41)
Everolimus + D	Ramalingam et al.	Single arm II	ORR 8%	Ramalingam et al. (42)
Erlotinib ± everolimus	Besse et al.	Randomized II	PFS 0.769 (0.506–1.167)	Besse et al. (43)
Buparlisib	BASALT-1	Single arm II	12 week PFS 23.3% (9.9–42.3)	Vansteenkiste et al. (44)
D ± PX-866	Levy et al.	Randomized II	med PFS 2 versus 2.9 mo (*p* = 0.65)	Levy et al. (45)
			med OS 7.9 versus 9.4 mo (*p* = 0.9)	
**Rb1/CDK**				
Palbociclib	Gopalan et al.	Single arm II	ORR 0%, SD 50% (8/16)	Gopalan et al. (46)
			Med PFS 12.5 week	
Abemaciclib	Patnaik et al.	I	ORR 3%, DCR 49%	Patnaik et al. (47)
**DDR2**				
Dasatinib	Johnson et al.	Single arm II	DCR 43%, ORR 3%	Johnson et al. (48)
			Med PFS 1.36 mo	
			Med OS 11.4 mo	
Dasatinib + erlotinib	Haura et al.	I/II	DCR 62%, ORR 7%	Haura et al. (49)
			Med PFS 2.7 mo	
			Med OS 5.6 mo	
**MET**				
PL + TAX ± onartuzumab	Hirsch et al.	Randomized II	PFS HR 0.95 (0.63–1.43)	Hirsch et al. (50)
			OS HR 0.90 (0.55–1.47)	
Erlotinib ± tivantinib	Sequist et al.	Randomized II	PFS HR 0.81 (0.57–1.16)	Sequist et al. (51)
			OS HR 0.87 (0.59–1.27)	
Erlotinib ± onartuzumab	METLung	III	PFS HR 0.99 (0.81–1.20)	Spigel et al. (52)
			OS HR 1.27 (0.98–1.65)	
Erlotinib ± onartuzumab	Spigel et al.	Randomized II	PFS HR 1.09 (0.73–1.62)	Spigel et al. (53)
			OS HR 0.80 (0.50–1.28)	

*C, carboplatin; Cis, cisplatin; CR, complete response; D, docetaxel; DCR, disease control rate; G, gemcitabine; HR, hazard ratio; Med, median; ORR, objective response rate; OS, overall survival; P, pemetrexed; PD, progressive disease; PFS, progression-free survival; PL, platinum; PR, partial response; SD, stable disease; T, taxane; TAX, paclitaxel; TTP, time to progression; V, vinorelbine*.

*^a^ORR in pem versus all matuzumab containing arms*.

*^b^Represents number of patients with measured response as detailed*.

## Epidermal Growth Factor Receptor

EGFR TKIs improve outcomes for patients with lung cancer harboring activating *EGFR* mutations. While these mutations are commonly found in adenocarcinoma, women, Asians and light or never smokers (3, 5–10), they are rarely found in pure SCC with series reporting a rate in the range of 0–5% (13). Despite this, EGFR TKI have shown significant benefit compared to placebo in patients with advanced lung cancer (all genotypes) having progressed on first or second-line chemotherapy, including SCC (28–30). More recently, Soria et al. reported further advantage of afatinib over erlotinib in the treatment of advanced unselected SCC (including mixed NSCLC) in terms of both PFS (median 2.6 versus 1.9 months; HR 0.81, 95% CI 0.69–0.96, *p* = 0.0103) and OS (median OS 7.9 versus 6.8 months; HR 0.81, 95% CI 0.69–0.95, *p* = 0.0077) (30). Of note, patients were previously treated with first-line platinum doublet and had no prior EGFR TKI directed therapies.

Monoclonal antibodies directed against EGFR have also been investigated in this setting. For example, several trials explored the use of cetuximab in combination with chemotherapy in treatment naïve patients, including two phase III trials with conflicting results (31, 32). A meta-analysis reported a HR of 0.878 (95% CI, 0.795–0.969; *p* = 0.01) for overall survival favoring the use of cetuximab in all lung cancer subtypes (33). Necitumumab, a second-generation recombinant human IgG1 monoclonal antibody, has also shown minor improvements in PFS and OS when added to gemcitabine/cisplatin first-line in advanced SCC versus gemcitabine/cisplatin alone (HR OS 0.84, 95% CI 0.74–0.96; *p* = 0.01) (34). No predictive markers of benefit were identified, although *EGFR* copy number may be promising (54). Conversely, other agents such as matuzumab and panitumumab have failed to show a benefit (35, 36). Despite the low frequency of actionable mutations, SCC shows high rates of *EGFR* amplification and protein expression that could explain these results (55–57). To date, different trials have reported inconsistent results using these findings as predictive biomarkers for response to EGFR directed therapies and their significance remains controversial (58).

## Fibroblast Growth Factor Receptor

Genomic abnormalities in the *FGFR* pathway have also been frequently reported in various malignancies including SCC of the lung (59). Most of these aberrations are *FGFR* amplifications with reported rates ranging from approximately 10–25%, while mutations are present in approximately 0–8% of cases (14, 16–18). It is hypothesized that this family of transmembrane receptors participates in many cellular processes including cell survival, differentiation, migration, angiogenesis, tissue homeostasis and repair, and inflammation (60–62). Clinically, *FGFR* amplifications are associated with smoking history and worse prognosis in SCC (63). In recent years, multiple FGFR-directed molecules, including both selective and non-selective FGFR inhibitors, have been developed but remain investigational to date. In the phase III LUME-lung 1 trial, nintedanib, an oral multiple TKI targeting FGFR1–3, vascular endothelial growth factor receptor 1–3, PDGFR α and β, RET, FLT3, and Src family kinases, was investigated in combination with docetaxel after failure of first-line therapy versus placebo (37). Despite marginal improvement in PFS in the overall study population, OS benefit was limited to adenocarcinomas. Dovitinib, a multikinase inhibitor of FGFR1–3, VEGFR1–3, PDGFR β, c-KIT, and FLT3, investigated in a phase II trial of SCC lung cancers showed modest antitumor activity and acceptable toxicity profile with most common significant side effects including gastro-intestinal toxicity (nausea, diarrhea, and anorexia), skin rash, and fatigue (38). Selective FGFR inhibitors, such as FGFR1–3 and VEGFR2 inhibitor AZD4547 and pan-FGFR inhibitor BGJ398, remain largely investigational, as early phase trials have reported mixed results in terms of efficacy (39, 40) (NCT00979134, NCT02154490, NCT02160041, NCT01004224). Other agents such as lucitanib (64) (NCT01283945, NCT02109016), ponatinib (NCT01935336), Bay1163877 (NCT02592785, NCT01976741), ARQ087 (NCT01752920), and JNJ-42756493 (NCT02699606) are also in development. Most trials enrolled molecularly enriched populations according to *FGFR* amplification. To date, there is however no standardized method or cut-off for amplification status with significant heterogeneity across trials.

## PI3KCA

Alterations in the *PI3KCA* pathway have also been implicated in the development and progression of advanced lung cancer (14). Its activation, triggering downstream AKT and mammalian target of rapamycin signaling, has been linked to gene amplification and mutations, which are both found predominantly in SCC in the range of 35 and 3–15%, respectively (14, 15, 19–21). This pathway is also upregulated through inactivating mutations and loss of its negative regulator *PTEN* and rarely *via AKT* mutations (14, 21, 65). In response to various growth factors, PI3KCA-AKT-mTOR participates in many cellular functions including cell growth, proliferation, differentiation, motility, and survival (66). In preclinical models, cells harboring *PI3KCA* alterations present aggressive phenotype and express markers of epithelial-to-mesenchymal transition (67). Clinically, these aberrations are also linked to EGFR inhibitor resistance (68). Previously, multiple trials have investigated the use of everolimus, an mTORC1 inhibitor, with disappointing results (41–43). Currently, various newer agents targeting this pathway are in development including isoform-specific and pan-isoform PI3KCA inhibitors, AKT inhibitors, and dual PI3KCA-mTOR inhibitors. Buparlisib, an oral inhibitor of class I PI3K (α, β, γ, and d), showed disappointing response rates in a phase II trial meeting futility criteria despite enrichment for PI3KCA pathway activation positive tumors (44). In phase I trials of advanced solid tumors including NSCLC, pilaralisib, an oral pan-class I PI3K inhibitor, has shown acceptable toxicity profile both as a single agent and in combination with EGFR inhibitors with preliminary efficacy limited to monotherapy use (69, 70). PX-866, an irreversible pan-isoform inhibitor of PI3K, failed to show benefit in terms of PFS and OS in a randomized phase II trial in combination with docetaxel compared to placebo (45). Trials investigating other selective PI3K inhibitors such as taselisib (NCT02785913, NCT02389842, NCT02154490, NCT02465060) and pictilisib (NCT01493843, NCT02389842) are currently ongoing both as single agents and in combination with chemotherapy.

## Rb1 and Cyclin-Dependent Kinases (CDK)

The *Rb1* pathway is also commonly disrupted in various cancers. In association with D-type CDK, CDK4 and CDK6 promote cell cycle progression from the G1 to S phase *via* phosphorylation of the tumor suppressor *Rb1*. P16, a tumor suppressor protein encoded by *CDKN2A*, also influences this pathway through its negative regulation of CDK4 and CDK6, which ultimately causes inhibition of Rb phosphorylation. Once phosphorylated, Rb is rendered inactive, driving cells into synthesis thus contributing to oncogenesis. Deregulation of this pathway occurs as a result of various mechanisms in SCC including *CDKN2A* inactivation *via* promoter methylation, deletions, and mutations, *Rb* mutations and deletions, and *CDK* amplifications (14, 71–74). Furthermore, preclinical data suggest activity of CDK inhibitors in lung cancer xenograft models, and therefore, CDK4/6 inhibitors are currently under investigation for the treatment of advanced lung cancers (74). In a phase II trial, Gopalan et al. found no responses to palbociclib, a highly specific CDK4/6 inhibitor, in patients with advanced lung cancers and negative p16 expression by immunohistochemistry (46). Interestingly, approximately half of evaluable patients had stable disease (SD) suggesting treatment may induce replicative senescence. Abemaciclib, another CDK4/6 inhibitor, showed acceptable toxicity profile and preliminary efficacy in a phase I trial of multiple tumor types, including NSCLC (47). Further trials investigating these agents are currently underway (NCT02411591, NCT02450539, NCT02152631, NCT02079636, NCT02022982, NCT02389842, NCT02897375, NCT02785939).

## Discoidin Domain Receptor Tyrosine Kinase 2

Discoidin domain receptor tyrosine kinase 2 is a widely expressed receptor tyrosine kinase (RTK) in normal cells that is activated through its interaction with various types of extracellular matrix protein collagen. Once activated by ligand binding and phosphorylation, DDR2 has been shown to promote various cellular functions such as migration, differentiation, proliferation, and survival (75). This RTK has been proposed as a potential treatment target in various cancers. Sequencing data has in fact shown mutations in the kinase domain of *DDR2* in approximately 1–4% of SCC (16, 22). Furthermore, *in vitro* studies have also demonstrated that cells harboring these mutations are sensitive to silencing of DDR2 by RNA interference. Multikinase inhibitors have been found to have *DDR2* directed activity in cell lines (76). Dasatinib, a multikinase inhibitor that targets *BCR-ABL*, Src family, c-KIT, PDGFR-β, and ephrin receptor approved for the treatment of chronic myelogenous leukemia (CML), has been investigated for the treatment of NSCLC. Pitini et al. reported a case of a patient with *DDR2* mutated SCC who presented a nearly complete response following treatment with dasatinib for a concurrent CML (77). In a phase II trial, this agent demonstrated moderate clinical activity in patients with unselected treatment naive advanced NSCLC (48). Its use was however limited by significant toxicity, in particular pleural effusion. Notably, one patient responded markedly to treatment with four others showing prolonged SD, suggesting potential benefit in a subset of patients. Unfortunately, investigators failed to identify a predictive biomarker in this subpopulation of responders. Another phase II trial of dasatinib in combination with erlotinib in heavily pretreated NSCLC showed modest efficacy with two patients having PR, one with an *EGFR* mutated adenocarcinoma and one with SCC (49). It is however challenging to estimate the antitumor activity of dasatinib in this setting as responses are more likely related to erlotinib.

## Mesenchymal-to-Epithelial Transition

The proto-oncogene *MET* is disrupted in various cancers including NSCLC (78). It encodes a RTK that, once activated by its ligand hepatocyte growth factor, promotes downstream signaling *via* multiple pathways such as PI3KCA, AKT, signal transducer and activator of transcription 3, and mitogen-activated protein kinase (79). Various activating alterations in *MET* have been reported in NSCLC. For example, *MET* amplification has been reported in approximately 6–10% of SCC, while mutations, particularly in exon 14, are more common in adenocarcinomas (23). Once upregulated, MET signaling contributes to cell survival, invasion, migration, and proliferation (79). Clinically, *MET* amplification has been linked to *EGFR* TKI resistance and poor prognosis (80). Cells harboring alterations in this pathway were found to be responsive to MET inhibitors that are commonly used in other tumor types such as crizotinib and cabozantinib (81, 82). Several clinical trials have investigated various TKI with MET directed activity for the treatment of advanced NSCLC with disappointing results in the SCC subpopulation so far (50–53). For example, onartuzumab, a monoclonal antibody directed against MET, failed to show significant antitumor activity in a phase II trial in combination with platinum-doublet chemotherapy (50). Moreover, a phase III trial of onartuzumab in combination with erlotinib was terminated early due to futility in terms of its primary outcome (OS) despite selection of patients with positive MET expression by immunohistochemistry (52). Tivantinib, a small-molecule MET inhibitor, showed modest antitumor activity in combination with erlotinib in unselected NSCLC (51). In subgroup analysis, benefit was however mostly noted in *KRAS* mutated patients and the subsequent phase III trial enrolled only non-squamous histology (83). Finally, identifying responding subpopulations represents a significant challenge in the development of these agents. In fact, selection of patients across trials has been inconsistent, with no clear definition of *MET* enriched populations. Overexpression has been defined using various methods including protein overexpression by immunohistochemistry, gene copy-number gain, and amplification by fluorescent *in situ* hybridization. Despite these challenges, multiple MET-directed molecules are currently under investigation for advanced NSCLC, including SCC (NCT02499614, NCT02034981, NCT00585195, NCT02925104, NCT02414139, NCT02929290, NCT02296879, etc).

## Immune Therapy

In recent years, immunotherapy agents have elicited great interest for the treatment of several tumor types. Various immune checkpoint inhibitors including antibodies directed against cytotoxic T-lymphocyte associated protein 4, programmed cell death protein 1 (PD-1), and programmed death ligand-1 (PD-L1) are under investigation or approved for clinical practice, revolutionizing the approach to lung cancer treatment. Patients diagnosed with SCC in particular have benefited from these advancements, as alternative treatments are sparse, and they have higher mutation burden, which may be associated with benefit. Agents such as nivolumab, pembrolizumab, and atezolizumab have demonstrated improvement in survival outcomes in the second-line setting including in SCC (84–87). Furthermore, Pembrolizumab showed improvement both PFS and OS for patients with strongly PDL-1-expressing tumors treated in the first-line setting. This was however not the case for first-line nivolumab, another PD-1 inhibitor that used less restrictive PDL-1 selection, which had similar PFS and OS but not superior outcomes (88) (NCT02041533). Much like targeted agents, the selection of patients seems to be an important factor when choosing the best course of therapy. Unfortunately, a predictive biomarker to guide this decision is lacking with PD-L1 expression status, a promising biomarker for the selection of the subgroup likely to benefit from PD-1 and PD-L1 inhibiting drugs, having shown mixed results so far. For example, in the Checkmate 017 study of nivolumab in advanced pretreated SCC patients, PDL-1 expression was not predictive of benefit and even those without PDL-1 expression derived survival gain (84). Conversely, PD-L1 expression was predictive in the Checkmate 057 trial of nivolumab in a similar setting in non-squamous NSCLC (85). Finally, smoking status, a simple clinical characteristic, could also represent a possible predictive marker of response.

## Conclusion

SCC represents complex tumors with alterations in various interacting pathways (14). Despite the current wealth of available molecular data and a vast array of clinical trial results, multiple challenges remain in the development of targeted therapies for this cancer. One recurring obstacle is the definition of subgroups that derive optimal benefit from investigational agents. With the current understanding of NSCLC now refined according to molecular profiles, individual subpopulations represent rare tumor types limiting their accrual into traditionally designed clinical trials. The revolutionized classification of lung cancer therefore requires an equally novel approach to clinical trial design. In fact, a growing number of “master protocols” with innovative schemes such as “basket” and “umbrella” biomarker-driven trials have been completed or are currently underway (89, 90) (NCT01042379). The LUNG-MAP trial, one such biomarker-based master protocol, is currently ongoing in multiple centers (90). Enrolled patients with advanced SCC are assigned to treatment arms according to detected targetable mutations identified through a comprehensive genomic profiling platform. Targeted agents such as taselisib, palbociclib, talazoparib, ABBV-399, rilotumumab, and AZD4547 have been included in this study. Furthermore, patients without actionable mutations are included in immune therapy sub-studies investigating various immune checkpoint inhibitors such as nivolumab, ipilimumab, durvalumab, and tremelimumab. Considering the dismal prognosis of patients diagnosed with advanced SCC, a greater focus on drug development and clinical trials remains of upmost importance to improve outcomes in this disease.

## Author Contributions

SS researched data for review topic, drafted manuscript, and edited manuscript revisions. NL researched data for review topic and edited manuscript.

## Conflict of Interest Statement

SS: none, NL: none known (honoraria for CME from Pfizer, Merck; travel funding for CME from Astrazeneca).
